# Molecular Features Distinguish Gastric Cancer Subtypes

**DOI:** 10.3390/ijms19103121

**Published:** 2018-10-11

**Authors:** Valli De Re

**Affiliations:** Immunopathology and Cancer Biomarkers/Bio-Proteomics Facility, Centro di Riferimento Oncologico, IRCCS, 33081 Aviano, Italy; vdere@cro.it; Tel.: +39-0434-659-672; Fax: +39-0434-659-659

Gastric cancer (GC) is a leading cause of cancer deaths. However, analysis of its molecular and clinical characteristics has been complicated by histological and etiological heterogeneity. Adenocarcinoma can be subdivided into histological Lauren and World Health Organization (WHO) classifications, however, this information has not led to the development of histologic subtype-specific treatment options. One way to potentially improve treatment for GCs is to better understand the molecular pathogenesis of the disease as well as the contribution of *Helicobacter pylori* infection and host immune responses leading to the development of an integrated histological and molecular classification schemes for GC.

Over the last several years, two major and comprehensive studies have been published focusing on a molecular classification of GC, the Cancer Genome Atlas (TCGA) and the Asian Cancer Research Group (ACRG) networks. Both Alessandrini [1] and Tirino [2], assessed the most common molecular GC classifications reported over the last years and found the main targetable molecular drivers highlighted by these studies. Indeed, only trastuzumab (anti-HER2 monoclonal antibody) and ramucirumab (anti-VEGFR monoclonal antibody) have proven to be successful in treating advanced or metastatic GC and they are currently used as a standard of care, but new drugs, e.g., nivolumab (PD-1 immune check-point inhibitor) showed activity against MSH-high locally advanced GC. Although the patient’s condition improved by using these drugs, clinically, the practical use of the results remain is limited. Thus, GC remains one the most lethal diseases in the world with systemic chemotherapy still necessary to support its advanced stages. The hope is that combining histological and molecular classification will help identify more specific and limited in number biomarkers that consent to categorized patients who will really benefit from such therapies. With this aim Machlowska et al. [3], highlights the current status of prognostic molecular biomarkers that can be used in GC with a particular attention to those impacting on peritoneal spreading and neo-vascularization of the tumor, two of the most important mechanism by which tumors become more aggressive. In respect of the discovery of predictive tumor response markers in specific GC subset, Caggiari et al. [4], report that the characterization of a CDH1 haplotype is associated with improved survival in metastatic GC targeted by HER2 therapy. Indeed, the positivity for the HER2 status (by IHC or by fluorescence in situ hybridization) is a prerequisite for trastuzumab (anti-HER2 monoclonal antibody), which is the treatment currently used as a standard of care in advanced and metastatic GC. However, due to the heterogeneity of the tumor, the identification of the targeted HER2 molecules the treatment is not sufficient to predict drug response. In the present study, the authors also proposed a functional role of E-cadherin, the protein codified by the CDH1 gene in the HER2 pathway, which may be involved in better response to treatment. Although, the role of the CDH1 mutations in this context requires elucidation through further studies, the study is of interest for its novelty and for its potential utility in selecting patients who may benefit from new anti-HER2 agents. Hyperfibrinogenemia is also an important risk factor known to influence GC development and outcome, but the exact fragment of fibrinogens preferentially produced in the tumor environment is less known. A study reported by Repetto et al. [5], provides evidence of an increase in region D of the fibrinogen B chain fragments, as well as the entire fibrinogen chain production, in the tumor biopsies of patients with GC compared to the equivalent biopsies at least 5 cm from the tumor lesion of the same patients. Furthermore, they found a relationship between the increase in the load of fibrinogen fragments in tumor mass with an increase of the platelets number with a higher GC stage and the stomach corpus localization. Their results, thus, sustain the potential role of fibrinogen and platelets in the progression of tumor cell growth and aggressiveness of the tumor. Their data support the usefulness of plasma fibrinogen/fibrinolysis evaluation and platelet count in GC to evaluate the treatment response and patients’ prognosis. Data have also helped to further characterize the interconnections between GC and platelet/coagulation pathways.

Part of the issue was focused on the characterization of specific GC subtypes to reinforce the TCGA classification. In this context Gullo et al. [6], investigate the transcriptomic landscape showed by EBV+ and the MSI-High subtypes identified in the TCGA classification. Their results strengthen the value for molecular segregation of these two subtypes and underline their relation with difference in clinical presentation. In particular, they confirm the importance of immunogenicity of EBV+ tumors and the mitotic signature of MSHI-high+ tumors, and reinforce the robustness of the Nanostring CodeSet proposed by TCGA classification. Notably, they highlighted a difference in the distribution of important checkpoint molecules, e.g., PD-1/PD-L1 molecules between EBV+ and MSHI-High subtypes, underling the best predictive value of protein expression rather than PD-L1 expression by immunohistochemistry to select GC patients eligible for anti-PD-1 immunotherapy. Their study offers a biological rationale to explain the unexpected positive response observed in patients harboring PD-L1-negative tumors, treated with anti-PD-1 therapy and a reasonable use of multiple immune targeted therapies in the specific EBV+ GC subtype, although further studies are necessary to sustain this proposal. The review reported by Dolcetti et al. [7], highlighted the observations of more recent advances in immunotherapeutic approaches overall of the GC subtypes. Indeed, immunotherapeutic approaches are still in the early phases but rapidly evolving in clinic. They discuss more important clinical trials reported by scientific communities, highlighting salient critical factors and possible solutions when they can be hypothesized. They also bring attention to the developments of more promising immunotherapies using adoptive cell and/or engineered cell; immune checkpoint inhibitors, immune modulator pathways, agonistic antibodies for co-stimulatory receptors, and cancer vaccines, thereby highlighting that for good clinical trials, the evaluation are also required to predict which patients will be responsive to particular treatments. Aldinucci et al. [8], focus on a particular immune axis: The interactions of chemokine CCL5, also known as RANTES, with its receptor CCR5, which regulates the immune and inflammatory responses by inducing lymphocytes and monocytes migration. Some researchers have demonstrated that cancer cells subvert the normal chemokine role, transforming them into a fundamental constituent of the immunosuppressive tumor microenvironment with tumor-promoting effects. The authors discuss the potential role of CCL5 leading to GC cell proliferation and metastasis and its proangiogenic effect in GC, although the exact functions of CCL5 in GC biology are not completely know. Moreover, CCR5 is not only a chemokine but also a co-receptor for HIV cell entry and a target of several drug researches, including GC treatment in pre-clinical and clinical trials as discussed by the authors. Notably, *H. pylori* increases the CCL5 secretion and interfere with the interaction between tumor cells and tumor microenvironment. Moreover, it was demonstrated that an increase in the CCL5 serum level and/or immunohistochemical staining is associated with more advanced GC stages and risk for peritoneal metastatization. Different algorithms include a CCL5 factor in order to predict treatment response, prognosis, and survival outcomes in GC. Thus, based on current knowledge, the CCL5/CCR5 axis may be considered a therapeutic target in GC.

Other landmark approaches to analytically photograph GC at molecular level are the modern mass spectrometry molecular imaging (MALDI-MSI) and flow cytometry. By using the first technique some pathologically significant molecules have already showed promise in the study of GC, providing greater insights into the molecular aspects of the disease and aiding in the identification of candidate biomarkers. Smith et al. [9] provided an overview of the MALDI-MSI innovative methodologies and summarize how the technique has been used to advance GC research for biomarker detection and for monitoring treatment response. Examples of MALDI-MSI applications in GC are the fasudil drug and its metabolites and inhibitors of the ROCK protein kinases, that can reach cancer cells in mice non-selectively. The proteomic differences may highlight a phenotypic tumor heterogeneity, which cannot be uncovered by using traditional histology; the identification is then successfully validated by immunohistochemistry of prognostic factors able to distinguish patients between stage I GC from those at the other stages; the identification of a protein profile predicting the HER+ GC tumor status with an accuracy of about 88–90%; the importance of difference in the distribution of lipids, metabolites and glicosilated fragments between the tumoral lesion and the non-neoplastic mucosa of a same patient. The second approach, the flow cytometry, has been used by Bockerstett [10], to analyze individually viable epithelial cells from gastric mucosa, which usually is limited due to difficulties in tissue processing. They develop and herein report an effective method for processing stomach tissue by enzymatic digestion and then analyze, via flow cytometry gastric epithelial cell, changes at single cell level from a large cell number of viable gastric cells in a model of inflammation induced gastric atrophy in mice. This approach results particularly useful for studying the inflammatory changes in surface markers on gastric epithelial cells during chronic disease. Their method confirms the up-regulation of MHC-II molecules on the epithelial cells caused by H-pylori-mediated inflammation. It is also possible that in the near future, a similar flow cytometric non-invasive diagnostic approach will be used to identify specific GC biomarker subtypes in circulating tumor cells.

The second part is in relation to environments and genetic factors that are known to increase the risk for GC development and how these factors may be useful to identify particular subjects that could be included in specific GC subtypes or at high risk for GC development.

*H. pylori* is the most abundant bacterium in the gastric epithelium and its presence was clearly associated with the risk of developing GC. In the last 100 years, infections have gradually declined due to new technologies, although other bacteria have been now identified in the stomach. Li and Perez. [11] discussed the potential role of the human gastric microbiota change in the presence or absence of *H. pylori* and moreover, they discuss which factors contribute to the increasing risk of GC. In particular, they confirm that the increased risk for GC is associated with the presence of highly virulent *H. pylori* strains (e.g., CagA+ and VacA+), and simultaneously by host genetic polymorphisms in the pro-inflammatory cytokine genes. However, it is now evident that during the progression of disease from *H. pylori* infection to GC, the stomach increases its pH thereby reducing the presence of *H. pylori*. In the same way, other bacteria increase with the possibility that the phenomena could be at patch trough the overall the stomach and with a different clinical outcome. Indeed, the change in the microbiota composition may also change the chronic inflammatory status in the stomach and a “point of no return” was reported in the cascade of events that lead to GC, which is associated with patients having intestinal metaplasia and dysplasia and is independent of *H. pylori* status. An elegant model to sustain gastric microbiota in the development of GC was in fact shown using the transgenic insulin-gastrin mouse model, but this could not be sufficient to demonstrate a direct role of microbiota in carcinogenesis. Nonetheless, the authors emphasize that in some regions, despite the decline of *H. pylori* infections, an increased incidence of GC was especially found in young adults (<40 years), GC diffuse type, and with no difference in the sex frequency. For the authors these new epidemiological data are particularly important since they could imply that changes in the gastric microbiota associated with new standards of living may be implicated in the specific increase in the GC development showed. On the other hand, the increase of GC incidence may be associated with the increase of another disease, like autoimmune gastritis that was found similarly to GC increase in the same population. Thus, a direct role for microbiota in GC development need further studies before being clearly accepted as a model of GC carcinogenesis. Kidane [12], discusses current molecular mechanisms that lead to DNA single and double-strand breaks and that reduce the capacity of DNA repair caused by *H. pylori* infection. The model discussed highlights the necessity for *H. pylori*-gastric cell contact and the infiltration of immune cells into the tumor microenvironment. The production of RONS, reactive oxygen species and nitrogen species, which cause DNA base damage and activation of the NF-κB factors that induces the cleavage of promoter gene regions and double strand breaks are the major consequence of *H. pylori* infection, although *H. pylori* itself may result directly from mechanisms not yet fully known through epigenetic alterations and overall a host genome instability. Excision DNA repair are complex and may be resumed in three major pathways that use different enzymes and recognition process: The base excision repair (BER) that use specific glycosylases and preferentially recognize small damages, the nucleotide excision repair (NER) involved also in bulky DNA reparation, and the DNA mismatch repair (MMR). BER, repairs during the cell cycle the majority of break damages resulting from oxidation and alkylation and it is the primary repair pathway occurring during *H. pylori* infection. Authors discuss how *H. pylori* is involved in BER and NER processes and the effect known today in enzyme alterations involved in these processes, including gene mutations occurring in the host and associated with the process of DNA repair. Authors also evidence that *H. pylori* infection enhances the transcription factor NF-κB pathway in immune and epithelial cells, thus resulting of the modulation of many DNA repair genes and the production of the inducible inflammatory mediator nitric oxide synthase (iNOS), which through the production of nitric oxide, contribute to enhanced inactivation of DNA repair enzymes and DNA double strand breaks. Thus, we can conclude that host genetic variants involved in DNA repair could modify the process of carcinogenesis in *H. pylori* infected hosts in any way, but that specific association among them require further studies. Indeed, the molecular mechanisms of DNA break formation, how these breaks are repaired and the interference of *H. pylori* in these processes remain largely to be clarified. Reprimo is a family of gene downstream effectors of p53-induced cell cycle arrest at G2/M checkpoint. Epigenetic silencing of RPRM, mainly by DNA methylation of its promoter region or P53 pathway, occurs at early stages and is a common event in GC. Amigo et al. [13], emphasize the role of this poorly studied gene in GC carcinogenesis. Of particular interest, previously authors demonstrated that methylation of the reprimo promoter region was associated with the infection of *H. pylori* and in particular with the more virulent cytotoxin-associated gene A (CagA) positive strains and that DNA methylation of reprimo may predict the progression of gastric lesions with a high sensitivity and specificity. Authors propose that reprimo methylation of cell-free DNA could be a marker for non-invasive discovery of GC in the next future.

While intestinal GC is more associated to *H. pylori* infection, the diffuse type composed by non-cohesive cells is more observable in a hereditary form. Ansari et al. [14], focus their review on the pathogenicity of this specific diffuse-GC type and report the most current understanding of the host factors, as well as the bacterial *H. pylori* factors that have been specifically involved. Although the pathogenicity of DGC has not yet been clarified in detail, authors indicate the central role of E-cadherin and cell-signaling pathways in the maintenance of cell integrity and function in particular in this subtype of GC. Melo et al. [15], highlight the state of art regarding the best methodologies, including the evaluation of migration dynamics of cells carrying E-cadherin variants in a transgene drosophila melanogaster model, to categorize the missense mutations in the CDH1, the gene codifying for the E-cadherin protein. Indeed, in the hereditary form of GC 155 different mutations have been reported to date but in about 17% of these cases the mutation remains with a function not predictable. The definition of how these alterations could perturb the expression and function of E-cadherin, as well as related signaling and cellular mechanisms, are fundamental to help clinicians and genetic counsellors in the management of the patients with GC and their familiars. Moreover, some mutations in the CDH1 gene may result in a slight down regulation rather than a complete abolition/function of the E-cadherin, and these alterations may be present also in other than hereditary form of GC. In that context, Melo and Seruca’s group is considered a worldwide reference center to study the predictable function of CDH1 mutations. Their studies are also relevant to gain further understanding of the GC pathogenesis. In that context, the study of Caggiari et al. [4], highlight the possibility that CDH1 gene mutations can also be used as a potential prognostic factor for GC survival.

Bizzaro [16] noted that autoimmune diseases may also predispose individuals to malignancies. A link between chronic autoimmune gastritis and GC development has been known from some time. Bizzaro et al. describes autoimmune gastritis and review its association with GC, in particular of the intestinal type and type I gastric carcinoid. The show particular attention to autoantibodies produced during autoimmune gastritis as markers for monitoring patient’s response to treatment and during follow-up. The low sensitivity of autoantibodies has limited their application in clinical practice for an early detection of patient at risk for GC, but in the next future, the availability of new multiplex technology for the simultaneous detection of many autoantibodies could to overcome these limitations. Another important autoimmune disease associated with the development of GC and lymphoproliferative disorders is the common variable immunodeficiency disorder (CVID), a hypogammaglobulinemia, highly variable and heterogeneous in clinical manifestations, although frequently expresses severe, recurrent, and chronic bacterial infections of the respiratory and gastrointestinal tracts. The exact molecular pathways underlying the relationships between CVID and GC remain poorly understood. Leone et al. [17] assessed the most frequent genetic abnormalities resulting in CIVD discovered today, although they account for less than 15% of the overall cases. They also provided a hypothetical mechanism of GC development based on the peculiar features of the tumor occurring in these patients. Accordingly, they propose a protocol to screening patients with CVID at risk for GC by using three easy and non-invasive tests based on the evidence of a megaloblastic or macrocytic anemia, a deficiency in serum vitamin B12 and iron, and a positive urea breath test for *H. pylori* infection. Another hereditable predisposition for GC is the Lynch syndrome (LS) and familial adenomatous polyposis (FAP), two autosomal dominant genetic conditions leading to the development of colorectal cancer, but also to other tumors. Fornasig et al. [18] reported detailed clinical and molecular features of GC occurring in these patients since characteristics of GC associated with these diseases are still not well known. There information’s added an important contribution to the recognition of patients at higher risk for GC development, which could be direct for the endoscopic surveillance. Family history of GC is a generic but a well-recognized risk factor for developing GC. Serum metabolic profiles including 188 serum metabolites were used by Corona et al. [19], to differentiate GC patients from first-degree relatives of patients with GC in two separate and independent series. The best discriminators they found belonged to phospholipids and acylcarnitines classes, and the discrimination increased in power when the C16 and M(OH)22:1 metabolites were integrated with serum pepsinogen-II value and with the age of the individual tested. The results of the study also provided new insights into the metabolism of GC. The increased acylcarnitines probably reflects alterations in the mitochondrial respiratory complex activities arising in GC that further increase *H. pylori* infection and the major age of patient with GC; while the decrease of some phosphatidylcholine lipid derivatives may be a consequence of a predisposition of tumor cells for a phospholipid storage. Authors propose that the effect of this storage may alter the cell lipid raft known to be involved in several tumor processes and at the same it may reflect the increase in tumor nerve growth observed in GC.
